# Multimodal structural disease progression of retinitis pigmentosa according to mode of inheritance

**DOI:** 10.1038/s41598-019-47251-z

**Published:** 2019-07-24

**Authors:** Ruben Jauregui, Vitor K. L. Takahashi, Karen Sophia Park, Xuan Cui, Julia T. Takiuti, Jose Ronaldo Lima de Carvalho, Stephen H. Tsang

**Affiliations:** 1 Department of Ophthalmology, New York-Presbyterian Hospital, New York, NY USA; 2 Jonas Children’s Vision Care and Bernard & Shirlee Brown Glaucoma Laboratory, New York, NY USA; 3 Weill Cornell Medical College, New York, NY USA; 4 Department of Ophthalmology, Federal University of São Paulo, São Paulo, Brazil; 5 Division of Ophthalmology, University of São Paulo Medical School, São Paulo, Brazil; 6 Departament of Ophthalmology, Empresa Brasileira de Servicos Hospitalares (EBSERH) - Hospital das Clinicas de Pernambuco (HCPE), Federal University of Pernambuco (UFPE), Recife, Brazil; 7 Department of Pathology & Cell Biology, Stem Cell Initiative (CSCI), Institute of Human Nutrition, College of Physicians and Surgeons, Columbia University, New York, NY USA

**Keywords:** Outcomes research, Retinal diseases

## Abstract

We analyze disease progression in retinitis pigmentosa (RP) according to mode of inheritance by quantifying the progressive decrease of the ellipsoid zone (EZ) line width on spectral domain optical coherence tomography (SD-OCT) and of the dimensions of the hyperautofluorescent ring on short-wave fundus autofluorescence (SW-FAF). In this retrospective study of 96 patients, average follow-up time was 3.2 ± 1.9 years. EZ line width declined at a rate of −123 ± 8 µm per year, while the horizontal diameter and ring area declined at rates of −131 ± 9 µm and −0.5 ± 0.05 mm^2^ per year, respectively. Disease progression was found to be slowest for autosomal dominant RP and fastest for X-linked RP, with autosomal recessive RP progression rates between those of adRP and XLRP. EZ line width and ring diameter rates of disease progression were significantly different between each mode of inheritance. By using EZ line width and horizontal diameter as parameters of disease progression, our results confirm that adRP is the slowest progressing form of RP while XLRP is the fastest. Furthermore, the reported rates can serve as benchmarks for investigators of future clinical trials for RP and its different modes of inheritance.

## Introduction

Retinitis pigmentosa (RP) refers to a heterogeneous group of rod-cone retinal dystrophies with an estimated prevalence estimated of around 1 in 4,000 people worldwide^1–3^. Patients classically present with a history of nyctalopia and problems with dark adaptation, followed by progressive visual field constriction^1,2^. At a cellular level, these symptoms arise due to a primary genetic defect in the rod photoreceptors, whose degeneration is associated with secondary cone cell death and eventual blindness in patients^1,4,5^. RP is a Mendelian disease that is most commonly inherited in an autosomal recessive (arRP) (50–60% of cases), autosomal dominant (adRP) (30–40%), or X-linked (XLRP) (5–15%) manner^1^. Studies have shown that among the three forms of RP, adRP typically presents with the mildest form of the disease while XLRP presents with the most severe^2,6^.

Spectral domain optical coherence tomography (SD-OCT) and short-wavelength fundus autofluorescence (SW-FAF) are two non-invasive imaging techniques traditionally used to monitor structural disease progression in RP patients over time. SD-OCT scans are obtained to analyze the EZ line width, as the point where it disappears delineates healthy and unhealthy retina and corresponds to the boundaries of the patient’s visual field^7–10^. As disease progresses, the EZ line shortens, making SD-OCT scans an important imaging modality to track disease progression. The signal (488 nm excitation) for SW-FAF arises from retinal pigment epithelium (RPE) lipofuscin, a product formed in photoreceptors from of all-*trans*-retinal reactions^11–13^. RP patients often exhibit a ring of hyperautofluorescence whose inner border corresponds to the lateral end of the EZ line on SD-OCT^8,14^. With disease progression, there is a proportional constriction of the hyperautofluorescent ring and shortening of the EZ line.

In this study, we aim to quantitatively analyze disease progression in RP with structural measurements by monitoring the progressive decrease of the EZ line width on SD-OCT and the dimensions and area of the hyperautofluorescent ring on SW-FAF over time. Furthermore, we characterize the progression rates of these parameters for each mode of inheritance observed in RP: autosomal recessive, autosomal dominant, and X-linked recessive. With the advent of gene therapy as a treatment modality for inherited retinal dystrophies, it is crucial to characterize the progression of RP according to its different modes of inheritance, as it is well established that disease progression varies as a function of inheritance.

## Methods

### Patients and clinical examination

Patient selection and clinical examinations were performed in similar manner to previous studies from our group^10,15^. All study procedures were defined and informed patient consent was obtained as outlined by the protocol #AAAR0284 approved by the Institutional Review Board at Columbia University Medical Center. The study adhered to the tenets of the Declaration of Helsinki. None of the data presented in this study, imaging, and genetic testing results are identifiable to individual patients. A retrospective review of 400 patients with a clinical diagnosis of RP that visited our clinic within the last two years was conducted at the Department of Ophthalmology at Columbia University. The clinical diagnosis was made by a retinal dystrophies specialist (SHT) based on presenting symptoms, family history, fundus examination, and full-field electroretinography (ffERG) and subsequently supported by clinical imaging and/or genetic testing. The inclusion criteria for this study were the diagnosis of RP along with clear media and adequate fixation to allow for high-quality imaging. In addition, each patient was screened for a history of long-term follow-up in our office, defined as having two visits at least 1 year apart, with each visit consisting of a complete ophthalmic examination. Ophthalmic examinations included a slit-lamp and dilated funduscopic examination, best-corrected visual acuity (BCVA), fundus autofluorescence (FAF, 488 nm excitation), and spectral domain optical coherence tomography (SD-OCT). Imaging across all modalities was conducted after pupil dilation (>7 mm) with phenylephrine hydrochloride (2.5%) and tropicamide (1%). Horizontal foveal SD-OCT scans measuring 9 mm and fundus autofluorescence (FAF, 488 nm excitation) were acquired with the Spectralis HRA + OCT (Heidelberg Engineering, Heidelberg, Germany). The FAF scans were acquired with either a 55 or 30-degree field of view. The exclusion criteria precluded patients affected by any other ocular disorder in addition to RP or patients without genetic characterization of their disease. Because the performed measurements may be correlated between the two eyes of a single patient, one eye from each patient was chosen for analysis based on inclusion/exclusion criteria, ensuring that each data point could be assumed to be independent from each other.

### Image analysis

Measurements of the horizontal diameter and area of the hyperautofluorescent ring on the SW-FAF imaging, along with the width of the ellipsoid zone (EZ) line from the SD-OCT scans, were acquired at each clinic visit for each patient. To mitigate bias and error in the measurement of these parameters, the same scans from each clinic visit were analyzed by two independent graders (RJ and VKLT). The measurements were performed using a built-in measurement tool in the Spectralis HRA + OCT software. The external boundary of the ring, which is better defined than the internal boundary, was used as the borderline for the diameter and area measurements. The horizontal diameter was defined as the longest distance between the nasal and temporal borders of the ring.

### Statistical analysis

The statistical analyses were performed using the Stata 12.1 (StataCorp, College Station, Texas, USA) software. The Pearson correlation was calculated for the measurements of both independent graders (see Supplementary Table S1). Given the high correlation between the two graders, the average of the two values obtained from the graders was calculated and used for subsequent analysis. Statistical analysis included descriptive statistics for demographics, EZ line width, horizontal diameter, and ring area for both visits. The progression rates, defined as the difference in values obtained between the follow-up and baseline visits divided by the length of follow-up, were calculated for these parameters. One-sample Student’s t-test was used to determine whether the mean progression rates were different from 0. The group of RP patients was then sub-divided into cohorts by mode of inheritance, and unpaired Student’s t-tests were used to compare these parameters among the different cohorts. Statistical significance was defined as a P-value less than 0.05.

## Results

### Patients

In total, 96 patients (96 eyes) with RP were analyzed for this study. Characteristics of the patients, including descriptive statistics for follow-up times and age at the time of visit are included in Table 1 (full genetic characterization of each presented patient, including genetic variants, are detailed in Supplementary Table S2). Among the 96 patients, 53 (55%) presented with arRP, 35 (36%) with adRP, and 8 (8%) with XLRP. From the arRP patient cohort, 6 patients presented with syndromic disease: 2 with Usher syndrome type 1 (caused by *MYO7A*) and 4 with Usher syndrome type 2 (3 with disease caused by *USH2A* and 1 with *GPR98*). The mean follow-up time was 3.2 ± 1.9 (SD) years with a median of 2.5 years for the entire RP cohort. The mean age during visit 1 for the entire cohort was 40.2 ± 18.9 years and 43.4 ± 19.3 years during visit 2. Genetic characterization and disease-causing variants were identified in all 96 patients. The most common disease-causing genes were *USH2A* in arRP (28.3%), *RHO* in adRP (37.1%), and *RPGR* for XLRP (100%) (Table 2).

**Table 1:** Descriptive statistics for age and follow-up time of the patient cohort sub-divided by mode of inheritance.

Patient Cohorts	N (%)	Age During Visit 1 (yr)	Age During Visit 2 (yr)				
RP total	96 (100)	40.2 ± 18.9	43.4 ± 19.3				
arRP	53 (55)	40.6 ± 18.6	43.4 ± 19.1				
adRP	35 (37)	43.9 ± 18.6	47.8 ± 18.3				
XLRP	8 (8)	21.7 ± 12.8	23.9 ± 12.6				
**Follow-up time** (**yr**)	**Mean**	**Standard Deviation**	**Quantile**				
			**Minimum**	**25** ^**th**^	**Median**	**75** ^**th**^	**Maximum**
RP total	3.2	1.9	1.0	1.8	2.5	4.0	8.1
arRP	2.8	1.5	1.0	1.8	2.3	3.5	7.4
adRP	3.8	2.2	1.1	1.9	3.0	5.5	8.1
XLRP	2.3	1.6	1.1	1.3	1.5	2.7	5.8

Data are summarized as mean ± standard deviation where appropriate. N = number; RP = retinitis pigmentosa; arRP = autosomal recessive; adRP = autosomal dominant; XLRP = X-linked recessive.

**Table 2:** Genetic characterization of the patient cohort sub-divided by mode of inheritance.

Mode of Inheritance	N	Genes with disease-causing variants (N)
arRP	53	*USH2A* (15)*, *PDE6β* (6), *EYS* (5), *PDE6α* (4), *CDHR1* (2), *CNGB1* (2), *DHDDS* (2), *KIZ* (2), *MAK* (2), *MERTK* (2), *MYO7A* (2)*, *C21ORF2* (1), *CERKL* (1), *FAM161A* (1), *GPR98* (1)*, *IFT140* (1), *NPHP1* (1), *REEP6* (1), *SPATA7* (1), *TULP1* (1)
adRP	35	*RHO* (13), *RP1* (9), *PRPF31* (4), *KLHL7* (3), *IMPDH1* (2), *GUCA1B* (1), *NRL* (1), *PRPF8* (1), *PRPH2* (1)
XLRP	8	*RPGR* (8)

N = number; RP = retinitis pigmentosa; arRP = autosomal recessive; adRP = autosomal dominant; XLRP = X-linked recessive. * denotes patients with syndromic arRP: 2 patients presented with Usher syndrome type 1 caused by *MYO7A* and 4 with Usher syndrome type 2 caused by *USH2A* (3) and *GPR98* (1).

### Rates for the parameters of disease progression

**Figure 1:**
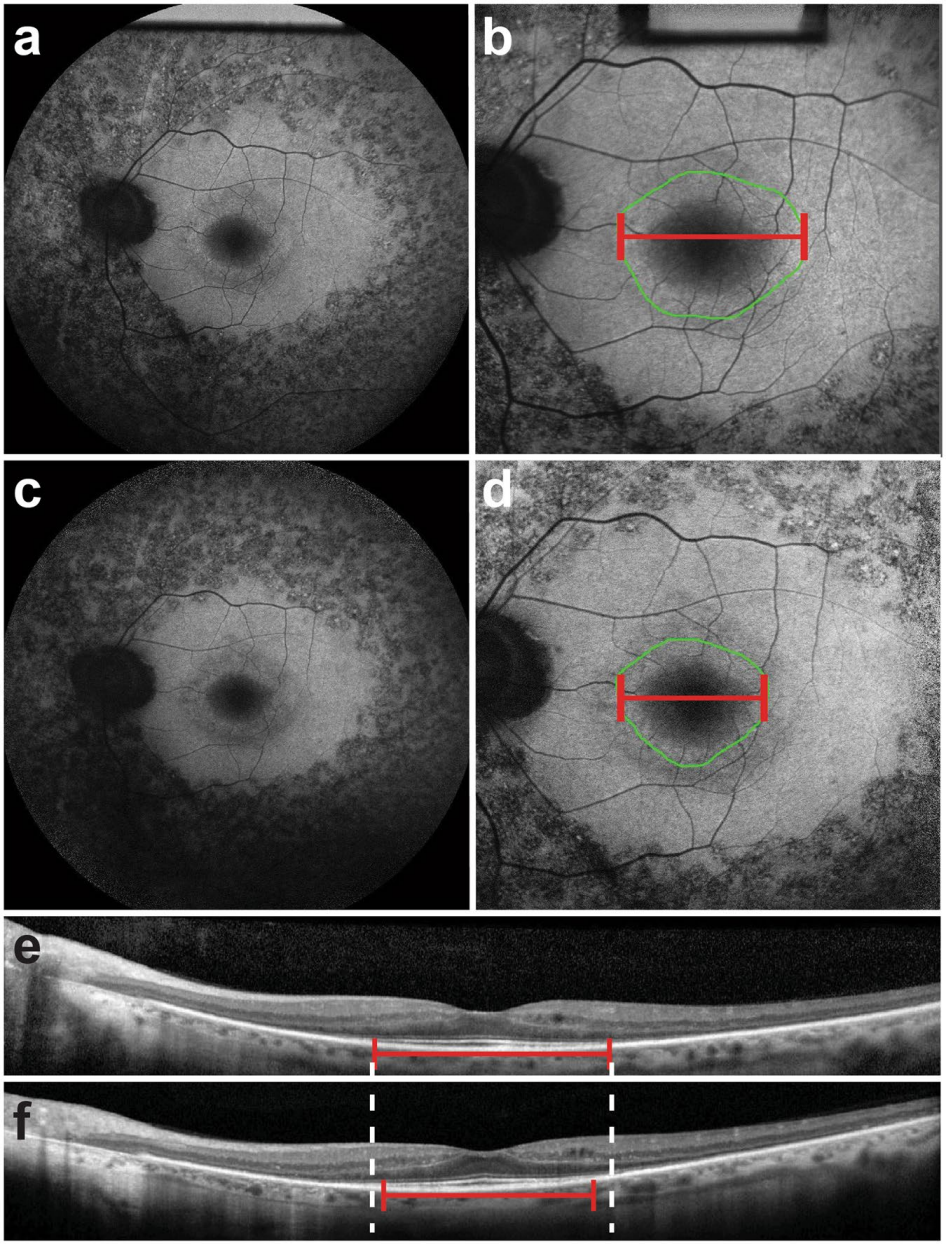
Progressive changes in short-wave fundus autofluorescence imaging and spectral domain optical coherence tomography scans of a patient with RP1-autosomal dominant retinitis pigmentosa. Short-wave fundus autofluorescence (SW-FAF) images with a 55- (**a**) and 30-degree (**b**) field of view during the first clinic visit of a patient with autosomal dominant retinitis pigmentosa (adRP) caused by the RP1 gene. The corresponding spectral domain optical coherence tomography (SD-OCT) scan is also shown (**e**). On the SW-FAF images, the area of the hyperautofluorescent ring is outlined in green (9.2 mm^2^), whereas the horizontal diameter is indicated by the red line (3993 µm). On the SD-OCT scans, the ellipsoid zone (EZ) line width is also marked with a red line (2435 µm). On the follow-up visit 6 years later, the EZ line shortened to 2080 µm (**f**), while both the ring area and horizontal diameter on SW-FAF (**d**) also decreased to 5.8 mm^2^ and 2080 µm, respectively.

**Figure 2:**
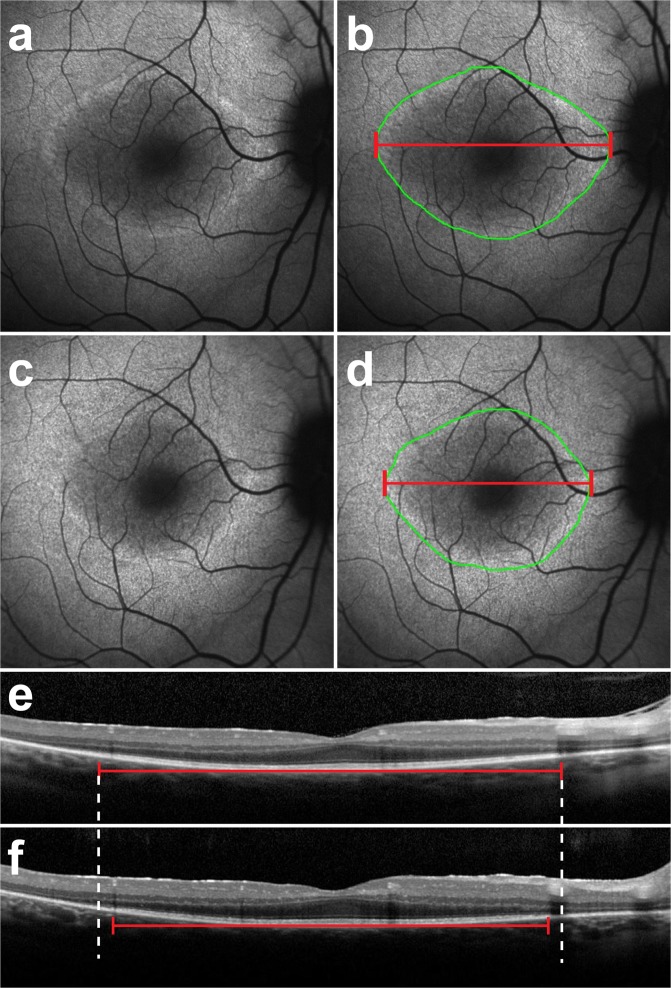
Progressive changes in short-wave fundus autofluorescence imaging and spectral domain optical coherence tomography scans of a patient with RPGR-mediated X-linked retinitis pigmentosa. Short-wave fundus autofluorescence (SW-FAF) images with a 30-degree field of view (**a**) during the first clinic visit of a patient with X-linked retinitis pigmentosa (XLRP) caused by the RPGR gene. The corresponding spectral domain optical coherence tomography (SD-OCT) scan is also shown (**e**). On the SW-FAF images, the area of the hyperautofluorescent ring is outlined in green (13.4 mm^2^), whereas the horizontal diameter (5057 µm) is indicated by the red line (**b**,**d**). On the SD-OCT scans, the ellipsoid zone (EZ) line width is also marked with a red line, measuring 4178 µm. On the follow-up visit 1.4 years later, the EZ line shortened to 3859 µm (**f**), while both the horizontal diameter and ring area on SW-FAF (**d**) decreased to 4668 µm and 11.8 mm^2^, respectively.

We observed a progressive decrease in each measured parameter: EZ line width and the horizontal diameter and area of the hyperautofluorescent ring (Table 3). For the collective cohort of RP patients, the EZ line width decreased at a rate of −123 ± 8 µm per year (P < 0.001), the horizontal diameter decreased at a rate of −131 ± 9 µm per year (P < 0.001), and ring area decreased at a rate of −0.5 ± 0.05 mm^2^ per year (P < 0.001). When patients were stratified by mode of inheritance, we observed distinct variations in each measure of disease progression (Table 3). In particular, patients with adRP exhibited the slowest disease progression in terms of decreases in EZ line width (−95 ± 11 µm/year, P = 0.043, <0.001) and horizontal diameter (−90 ± 10 µm/year, P = 0.001, <0.001) when compared to patients with arRP and XLRP (Fig. 1). Contrarily, patients with XLRP exhibited the fastest disease progression in regards to EZ line width (219 ± 31 µm/year) and horizontal diameter (−243 ± 45 µm/year) (Fig. 2). While the ring area in adRP patients decreased the slowest (−0.5 ± 0.07 mm^2^/year) in comparison to patients with XLRP (−0.7 ± 0.19 mm^2^/year), no statistically significant differences were found among the different modes of inheritance (Table 4).

**Table 3:** Progression rates for ellipsoid zone (EZ) line width, horizontal diameter and ring area of the hyperautofluorescent ring observed in the cohort of retinitis pigmentosa patients sub-divided by mode of inheritance.

Patient Cohort	Progression rates per year					
	EZ line width (µm)	P-value*	Ring diameter (µm)	P-value*	Ring area (mm^2^)	P-value*
RP total	−123 ± 8	<0.001	−131 ± 9	<0.001	−0.5 ± 0.05	<0.001
arRP	−128 ± 11	<0.001	−140 ± 11	<0.001	−0.6 ± 0.07	<0.001
adRP	−95 ± 11	<0.001	−90 ± 10	<0.001	− 0.5 ± 0.07	<0.001
XLRP	−219 ± 31	<0.001	−243 ± 45	0.001	−0.7 ± 0.19	0.006

Data are summarized as mean ± standard error where appropriate. EZ = ellipsoid zone; RP = retinitis pigmentosa; arRP = autosomal recessive; adRP = autosomal dominant; XLRP = X-linked recessive. *Calculated using one-sample Student’s t-test to test for a difference from 0.

**Table 4:** P-values from statistical analyses comparing the progression rates of the ellipsoid zone (EZ) line width, horizontal diameter and ring area of the hyperautofluorescent ring among the different inheritance modes of retinitis pigmentosa.

	EZ line width*	Ring diameter*	Ring area*	
arRP	0.043	0.001	*0*.*436*	adRP
adRP	<0.001	<0.001	*0*.*165*	XLRP
XLRP	0.003	0.002	*0*.*452*	arRP

EZ = ellipsoid zone; RP = retinitis pigmentosa; arRP = autosomal recessive; adRP = autosomal dominant; XLRP = X-linked recessive. *P-values were calculated using the two-sample Student’s t-test to test for a difference among the two groups. Results that are not statistically significant are italicized.

## Discussion

In this study, we use the structural variables of EZ line width and constriction of the hyperautofluorescent ring to characterize the progression of RP, both as a whole and per its different modes of inheritance. Although multiple studies have analyzed disease progression in RP patients using these same variables, all studies investigate non-stratified patient populations that include autosomal recessive (arRP), autosomal dominant (adRP), and X-linked RP (XLRP). The lack of stratification based on mode of inheritance makes it impossible to quantify nuances in progression rates among arRP, adRP, and XLRP, despite the existing clinical knowledge that RP progression varies among these three inheritance forms^16,17^.

In contrast to the patient cohorts of previous RP progression studies, our large sample size (96 patients) with complete genetic characterization allow us to observe a significant difference in progression rates that encompasses a more comprehensive body of RP patients. As a comparison, a study by Cabral *et al*. analyzed a cohort of 81 patients (41 patients with arRP, 24 with adRP, 4 with XLRP, and 12 with Usher syndrome) for an average follow-up time of 3.1 years^16^. Only 31 patients (40.3%) had genetic characterization. In another study by Sujirakul *et al*., a patient cohort composed of 71 patients (48 patients with arRP, 19 with adRP, and 4 with XLRP) was analyzed for an average follow-up time of 2.1 years, with only 26 genetically characterized patients (36.6%)^17^.

In our study, we report a yearly decrease of −123 µm for the EZ line width and a yearly decrease of −131 µm for the horizontal diameter for the entire RP cohort—rates that are comparable to those reported in previous studies^16,17^. Furthermore, our reported rates for each mode of inheritance support the notion that adRP is the mildest form of RP and XLRP is the most severe. When analyzing EZ line width as a parameter of progression, for example, we observe a rate of −95 µm per year for adRP, compared to a rate of −128 µm per year for arRP and −219 µm per year for XLRP. A similar trend is observed for the horizontal ring diameter and ring area. Nevertheless, we were not able to observe a significant difference in the ring area rate when comparing the different modes of inheritance. This suggests that measuring ring area as a parameter of progression is not as sensitive as measuring the EZ line width or the ring diameter. Of note, the average age of the XLRP group (21.7 and 23.9 years at visit 1 and 2, respectively) was younger than those of the adRP (45.9 and 49.6 years) and arRP (41.6 and 44.5 years) groups. Nevertheless, this is expected, as XLRP is the most severe form of RP; one study reported that the age of legal blindness is 32 years younger in XLRP patients than in adRP^6^. Thus, per our inclusion criteria, the addition of older XLRP patients was not often possible, given that it is difficult to obtain high-quality images from patients with advanced RP. Nevertheless, our reported EZ line width rate of progression for XLRP is similar to the reported rate of −248 µm per year in a previous study that analyzed XLRP disease progression^18^.

Other studies have used visual function parameters (e.g. visual acuity, Goldmann visual field areas, and 30 Hz cone ff-ERG amplitudes) to characterize the different modes of RP inheritance. In 2007, Sandberg *et al*. compared a cohort of 113 patients with *RPGR* variants causing XLRP to 134 patients with *RHO* variants causing adRP. They reported that *RPGR*-XLRP patients lose visual field and visual acuity more rapidly than those with *RHO*-adRP, although the rates of ERG loss were comparable between the two groups. In 2008, another study by Sandberg *et al*. compared 125 patients with *USH2A* mutations to the patients from the 2007 study that included *RHO*-adRP and *RPGR*-XLRP patients. They reported that *USH2A* patients lose visual acuity faster than *RHO* patients but slower than *RPGR* patients. In addition, they saw that *USH2A* patients lose visual field and ERG 30 Hz cone amplitudes faster than *RHO* and *RPGR* patients. Of note, the *USH2A* patient cohort included patients with both arRP and Usher syndrome type 2. The findings of a previous study, which suggest that cone function as measured by 30 Hz ERG is higher in non-syndromic as compared to syndromic *USH2A* patients^19^, may account for Sandberg *et al*.’s observation that the loss of 30 Hz ERG function was faster in *USH2A* patients compared to *RPGR*.

In conclusion, our study is the first to compare RP disease progression by using the structural parameters of EZ line width and hyperautofluorescent ring area and diameter among the different modes of RP inheritance. This study provides baseline progression rates that can be used by investigators to track the success of clinical trials, as constriction of the EZ line width and hyperautofluorescent ring are expected to provide meaningful endpoints for monitoring efficacy of treatment trials. Furthermore, these non-invasive retinal imaging methods are widely available and rapidly provide a direct and sensitive measure of disease progression and endpoints. Natural history of disease progression data will help inform the design of outcome measures used in the various upcoming gene therapy trials.

## Supplementary information

Supplementary Table 1 and 2

## Data Availability

The datasets generated during and/or analyzed during the current study are available from the corresponding author on reasonable request.
