# Supplementary material for: Multimodal structural disease progression of retinitis pigmentosa according to mode of inheritance
Source: Sci Rep. 2019 Jul 24;9:10712. doi: 10.1038/s41598-019-47251-z (PMC6656765; doi:10.1038/s41598-019-47251-z)
Supplement: Supplementary file 1 — Supplementary Table 1 and 2 [file 41598_2019_47251_MOESM1_ESM.pdf]

Multimodal structural disease progression of retinitis pigmentosa according to mode of inheritance

Ruben Jauregui [1,2,3], Vitor K. L. Takahashi [1,2,4], Karen Sophia Park [1,2], Xuan Cui [1,2], Julia T. Takiuti [1,2,5], Jose R. L. Carvalho-Jr [1,2,4,6], Stephen H. Tsang [1,2,7 §].

[1] Department of Ophthalmology, New York-Presbyterian Hospital, New York, NY, USA
[2] Jonas Children's Vision Care and Bernard & Shirlee Brown Glaucoma Laboratory, New York, NY, USA
[3] Weill Cornell Medical College, New York, NY, USA
[4] Department of Ophthalmology, Federal University of São Paulo, São Paulo, Brazil
[5] Division of Ophthalmology, University of São Paulo Medical School, São Paulo, Brazil
[6] Empresa Brasileira de Servicos Hospitalares (EBSERH) - Hospital das Clinicas de Pernambuco (HCPE), Departament of Ophthalmology, Federal University of Pernambuco (UFPE), Recife, Brazil.
[7] Department of Pathology & Cell Biology, Stem Cell Initiative (CSCI), Institute of Human Nutrition, College of Physicians and Surgeons, Columbia University, New York, NY, USA.
[§] Corresponding author

Running title: Structural disease progression in retinitis pigmentosa

Address Correspondence: Stephen H. Tsang, MD, PhD, Harkness Eye Institute, Columbia University Medical Center, 635 West 165th Street, Box 212, New York, NY 10032, Phone: (212) 342-1189 / Fax: 212-305-4987 / Email: sht2@cumc.columbia.edu

Supplementary Table 1. Correlations between the repeated measurements from both independent graders for horizontal diameter, vertical diameter, and ring area of the hyperautofluorescent rings at visit 1 and 2.

	**EZ line width**		**Horizontal diameter**		**Ring area**	
**Pearson correlation**	**Visit 1**	**Visit 2**	**Visit 1**	**Visit 2**	**Visit 1**	**Visit 2**
r	0.999	0.993	0.966	0.997	0.997	0.999
P-value	< 0.001	< 0.001	< 0.001	< 0.001	< 0.001	< 0.001

EZ = ellipsoid zone.

Supplementary Table 2. Complete characterization of the disease-causing genetic variants of the patients along with length of follow-up and best-corrected visual acuity at both visit 1 and visit 2.

Patient ID	Disease	Gene	Variants			Length of Follow-up (yrs)	Visit 1 BCVA	Visit 2 BCVA
			cDNA Change	Protein Change	Zygosity			
1	adRP	*RHO*	c.404G>T	p.R135L	*hetero*	3.7	20/30; 20/25	20/40; 20/40
2	adRP	*RHO*	c.568G>A	p.D190N	*hetero*	7.1	20/25; HM	20/40; HM
3	adRP	*RHO*	c.266G>A	p.G89D	*hetero*	7.1	20/125; 20/25	20/150; 20/40
4	adRP	*RHO*	c.316G>A	p.G106R	*hetero*	5.4	20/40; 20/40	20/50; 20/50
5	adRP	*RHO*	c.800C>T	p.P267L	*hetero*	8.1	20/20; 20/25	20/40; 20/40
6	adRP	*RHO*	c.102G>A	p.T342M	*hetero*	7.5	20/50; 20/40	20/50; 20/60
7	adRP	*RHO*	c.328T>C	p.C110R	*hetero*	4.2	20/50; 20/40	20/200; LP
8	adRP	*RHO*	c.937-27_-19delCCCTGACTC	n/a	*hetero*	5.6	20/20; 20/20	20/40; 20/40
9	adRP	*RHO*	c.568G>A	p.D190N	*hetero*	3.0	20/25; 20/20	20/40; 20/40
10	adRP	*RHO*	c.404_4005delinsGG>TT	p.R135L	*hetero*	1.7	20/300; 20/250	CF 3ft; 20/400
11	adRP	*RHO*	c.328T>C	p.C110R	*hetero*	2.8	20/30; 20/20	20/40; 20/25
12	adRP	*RHO*	c.50C>T	p.T17M	*hetero*	1.3	20/20; 20/20	20/30; 20/30
13	adRP	*RHO*	c.328T>C	p.C110R	*hetero*	2.8	20/20; 20/20	20/25; 20/25
14	adRP	*RP1*	c.2105_2108del	p.I702Tfs*10	*hetero*	1.1	20/20; 20/30	20/25; 20/30
15	adRP	*RP1*	c.5107delT	n/a	*hetero*	2.4	20/20; 20/20	20/25; 20/30
16	adRP	*RP1*	c.2105_2108del	p.I702Tfs*10	*hetero*	1.1	20/20; 20/20	20/25; 20/25
17	adRP	*RP1*	c.2285_2289delTAAAT	p.L762Yfs*17	*hetero*	6.5	20/30; 20/25	20/40; 20/30
18	adRP	*RP1*	c.2479G>C	p.E827Q	*hetero*	7.3	20/30; 20/30	20/40; 20/40
19	adRP	*RP1*	c.2029C>T	p.R677*	*hetero*	5.0	20/20; 20/20	20/25; 20/25
20	adRP	*RP1*	c.2285_2289delTAAAT	p.L762Yfs*17	*hetero*	3.7	20/25; 20/40	20/40; 20/50
21	adRP	*RP1*	c.2285_2289delTAAAT	p.L762Yfs*17	*hetero*	4.6	20/25; 20/30	20/30; 20/40

22	adRP	*RP1*	c.1437G>T	p.M479I	*hetero*	1.9	20/20;<br>20/25	20/20;<br>20/25
23	adRP	*PRPF31*	c.383T>A	p.L128*	*hetero*	4.6	20/25;<br>20/20	20/40;<br>20/25
24	adRP	*PRPF31*	c.420+6C>T	n/a	*hetero*	2.2	20/25;<br>20/25	20/25;<br>20/25
25	adRP	*PRPF31*	c.1222C>T	p.R408W	*hetero*	2.5	20/80;<br>20/150	20/150;<br>20/400
26	adRP	*PRPF31*	c.1066G>A	p.G356S	*hetero*	1.3	20/25;<br>20/50	20/50;<br>20/50
27	adRP	*KLHL7*	c.433A>G	p.N145D	*hetero*	6.9	20/30;<br>20/30	20/40;<br>20/40
28	adRP	*KLHL7*	c.472T>C	p.C158R	*hetero*	2.8	20/20;<br>20/25	20/30;<br>20/40
29	adRP	*KLHL7*	c.433A>G	p.N145Y	*hetero*	1.8	20/30;<br>20/30	20/30;<br>20/30
30	adRP	*IMPDH1*	c.962C>T	p.R321V	*hetero*	2.5	20/50;<br>20/70	20/60;<br>20/70
31	adRP	*IMPDH1*	c.1280C>T	p.P427L	*hetero*	1.1	20/40;<br>20/30	20/40;<br>20/60
32	adRP	*GUCA1B*	c.153_155delTGA	p.D51del	*hetero*	5.2	20/20;<br>20/20	20/40;<br>20/40
33	adRP	*NRL*	c.149C>T	p.S50L	*hetero*	6.1	20/25;<br>20/30	20/40;<br>20/40
34	adRP	*PRPF8*	c.6991delG	p.E2331fs	*hetero*	1.9	20/20;<br>20/30	20/25;<br>20/40
35	adRP	*PRPH2*	c.652T>C	p.S218P	*hetero*	1.8	20/25;<br>20/40	20/40;<br>20/50
36	arRP	*USH2A*	c.9371+A>G<br>c.11048-2A>G	n/a<br>n/a	*hetero*<br>*hetero*	1.0	20/20;<br>20/20	20/20;<br>20/20
37	arRP	*USH2A*	c.13378A>T<br>c.15178T>C	p.I4460L<br>p.S5060P	*hetero*<br>*hetero*	6.6	20/125;<br>20/40	20/400;<br>20/60
38	arRP	*USH2A*	c.10073G>A<br>c.920_923dupGCCA	p.C3358Y<br>p.H308Qfs	*hetero*<br>*hetero*	4.0	20/20;<br>20/20	20/30;<br>20/30
39	arRP	*USH2A*	c.12575G>A<br>c.7863delA	p.R4192H<br>p.2623Hfs*18	*hetero*<br>*hetero*	3.9	20/50;<br>20/25	20/50;<br>20/30
40	arRP	*USH2A*	c.10073G>A<br>c.9110G>A	p.C3358Y<br>p.R3037H	*hetero*<br>*hetero*	3.9	20/25;<br>20/25	20/30;<br>20/30
41	arRP	*USH2A*	c.12575G>A<br>c.12575G>A	p.R4192H<br>p.R4192H	*hetero*<br>*hetero*	2.1	20/30;<br>20/25	20/50;<br>20/50
42	arRP	*USH2A*	c.9676C>T<br>c.1478A>G	p.R3226*<br>p.Y493C	*hetero*<br>*hetero*	3.5	20/20;<br>20/20	20/30;<br>20/30
43	arRP	*USH2A*	c.12575G>A<br>c.2802T>G	p.R4192H<br>p.C934W	*hetero*<br>*hetero*	2.6	20/20;<br>20/20	20/25;<br>20/25
44	arRP	*USH2A*	c.13223T>C<br>c.13231C>G;<br>c.4251+1G>A	p.V4408A<br>p.L4411V;<br>n/a	*hetero*<br>*hetero*<br>*hetero*	2.4	20/30;<br>20/50	20/40;<br>20/60
45	arRP	*USH2A*	c.2276G>T	p.C759F	*homo*	1.4	20/20;<br>20/20	20/40;<br>20/30

46	arRP	*USH2A*	c.12575G>A<br>c.7595-2144A>G	p.R4192H<br>n/a	*hetero*<br>*hetero*	7.3	20/20;<br>20/20	20/30;<br>20/30
47	arRP	*USH2A*	c.2522C>A<br>c.5975A>G	p.S841Y<br>p.Y1992C	*hetero*<br>*hetero*	2.9	20/20;<br>20/50	20/70;<br>20/100
48	arRP/USH2	*USH2A*	c.3713C>G<br>c.9459C>A	p.T1238R<br>p.C3153*	*hetero*<br>*hetero*	2.9	20/40;<br>20/40	20/60;<br>20/60
49	arRP/USH2	*USH2A*	c.13577G>A<br>c.5775A>G	p.R4526Q<br>p.T1925T	*hetero*<br>*hetero*	4.9	20/20;<br>20/20	20/30;<br>20/30
50	arRP/USH2	*USH2A*	c.10712C>T<br>c.4711G>C	p.T3571M<br>p.A1571P	*hetero*<br>*hetero*	1.8	20/30;<br>20/50	20/50;<br>20/60
51	arRP	*PDE6β*	c.1927_1967del41	n/a	*homo*	1.8	20/20;<br>20/40	20/30;<br>20/50
52	arRP	*PDE6β*	c.1923_1969ins6del4<br>7	p.T641Tfs*31	*homo*	1.1	20/25;<br>20/25	20/25;<br>20/25
53	arRP	*PDE6β*	c.1923_1969ins6del4<br>7	p.T641Tfs*31	*homo*	3.2	20/20;<br>20/20	20/40;<br>20/40
54	arRP	*PDE6β*	c.1540delC	p.L514Wfs	*homo*	4.5	20/50;<br>20/40	20/60;<br>20/50
55	arRP	*PDE6β*	c.1488delC<br>c.1669C>T	p.T497Pfs*78<br>p.H557Y	*hetero*<br>*hetero*	2.9	20/60;<br>20/50	20/60;<br>20/60
56	arRP	*PDE6β*	c.1655G>A	p.R552Q	*homo*	5.3	20/100;<br>20/100	20/200;<br>20/200
57	arRP	*EYS*	c.6528C>A	p.Y2176*	*homo*	3.5	20/40;<br>20/50	20/50;<br>20/70
58	arRP	*EYS*	c.8111T>G<br>c.9316_9336delCCA<br>ATTTTGTTGGCAA<br>AATT	p.L2740*<br>p.T3106Kfs*13	*hetero*<br>*hetero*	2.8	20/50;<br>20/60	20/70;<br>20/70
59	arRP	*EYS*	c.4120C>T	p.R1374*	*homo*	1.3	20/20;<br>20/25	20/20;<br>20/30
60	arRP	*EYS*	c.1645G>T<br>c.2992+1G>A	p.E549*<br>n/a	*hetero*<br>*hetero*	3.9	20/25;<br>20/25	20/50;<br>20/50
61	arRP	*EYS*	c.4402G>C<br>c.3250A>C	p.D1468H<br>p.T1084P	*homo*<br>*homo*	2.0	20/200;<br>20/400	20/800;<br>20/800
62	arRP	*PDE6α*	c.304C>A<br>c.769C>T	p.R102S<br>p.R257*	*hetero*<br>*hetero*	1.8	20/150;<br>20/150	20/200;<br>20/200
63	arRP	*PDE6α*	c.304C>A<br>c.908C>G	p.R102S<br>p.S303C	*hetero*<br>*hetero*	1.6	20/50;<br>20/50	20/100;<br>20/100
64	arRP	*PDE6α*	c.304C>A<br>c.908C>G	p.R102S<br>p.S303C	*hetero*<br>*hetero*	2.9	20/40;<br>20/50	20/50;<br>20/60
65	arRP	*PDE6α*	c.1705C>A<br>c.2263C>T	p.Q569K<br>p.Q755*	*hetero*<br>*hetero*	1.0	20/25;<br>20/25	20/30;<br>20/30
66	arRP	*CDHR1*	c.2522_2528delTCT<br>CTGA	p.I841Sfs*119	*homo*	1.7	20/40;<br>20/40	20/60;<br>20/60
67	arRP	*CDHR1*	c.1463delG	p.G488fs	*homo*	2.1	20/50;<br>20/50	20/150;<br>20/150
68	arRP	*CNGB1*	c.3150delG	p.F105Lfs*12	*homo*	4.9	20/25;<br>20/25	20/40;<br>20/40
69	arRP	*CNGB1*	c.1896C>A<br>c.3150delG	p.C632*<br>p.G1050Gfs*13	*hetero*<br>*hetero*	1.4	20/40;<br>20/40	20/50;<br>20/50

70	arRP	*DHDDS*	c.124A>G	p.K42E	*homo*	3.9	20/25; 20/40	20/30; 20/40
71	arRP	*DHDDS*	c.124A>G	p.K42E	*homo*	1.1	20/30; 20/50	20/30; 20/70
72	arRP	*KIZ*	c.119_122delAACT	p.K10Ifs*14	*homo*	2.1	20/30; 20/80	20/50; 20/100
73	arRP	*KIZ*	c.226C>T	p.R76*	*homo*	2.2	20/40; 20/50	20/40; 20/50
74	arRP	*MAK*	c.1291A>T	p.K431*	*homo*	2.3	20/20; 20/25	20/30; 20/30
75	arRP	*MAK*	c.1297_1298ins	p.K433Rfs*31	*homo*	1.2	20/20; 20/25	20/25; 20/30
76	arRP	*MERTK*	c.1133C>T	p.T378M	*homo*	1.4	20/25; 20/25	20/30; 20/30
77	arRP	*MERTK*	c.2189+1G>T	n/a	*homo*	2.1	20/40; 20/40	20/50; 20/50
78	arRP/USH1	*MYO7A*	c.4544_4551delAGA TCATGinsCA	p.E1515_M1517 delinsA	*homo*	1.9	20/60; 20/40	20/80; 20/40
79	arRP/USH1	*MYO7A*	c.634C>T c.999T>G	p.R212C p.Y333*	*hetero* *hetero*	2.2	20/40; 20/50	20/60; 20/60
80	arRP	*C21ORF2*	c.670G>T	p.A224S	*homo*	4.0	20/50; 20/60	20/80; 20/150
81	arRP	*CERKL*	c.847C>T	p.R283*	*homo*	1.6	20/50; 20/60	20/50; 20/60
82	arRP	*FAM161A*	c.1321dupC	p.H441Pfs*15	*homo*	2.2	20/25; 20/40	20/30; 20/40
83	arRP	*GPR98*	c.9430G>T c.10769+2T>A	p.E3144* n/a	*hetero* *hetero*	2.2	20/40; 20/40	20/50; 20/50
84	arRP	*IFT140*	c.2788C>T c.3141+13C>T	p.H930Y n/a	*hetero* *hetero*	2.4	20/25; 20/80	20/40; 20/100
85	arRP	*NPHP1*	c.1078C>G c.1434+9G>A	p.L360V n/a	*hetero* *hetero*	7.4	20/50; 20/60	20/200; 20/200
86	arRP	*REEP6*	c.295G>A	p.E99K	*homo*	1.7	20/20; 20/20	20/30; 20/25
87	arRP	*SPATA7*	c.1100A>G c.1101_1102delTC	p.Y367C p.L368Efs*4	*hetero* *hetero*	3.5	20/25; 20/50	20/50; 20/100
88	arRP	*TULP1*	c.349G>A	p.E117K	*homo*	3.5	20/80; 20/50	20/100; 20/60
89	XLRP	*RPGR*	c.202G>A	p.G68R	*hemi*	5.8	20/20; 20/30	20/25; 20/40
90	XLRP	*RPGR*	c.2194del	p.E732Rfs*83	*hemi*	3.2	20/40; 20/40	20/40; 20/50
91	XLRP	*RPGR*	c.2752G>T	p.G918*	*hemi*	2.5	HM; 20/40	HM; 20/40
92	XLRP	*RPGR*	c.1059_1059+2delG GT	n/a	*hemi*	1.3	20/25; 20/25	20/30; 20/30
93	XLRP	*RPGR*	c.1307G>A	p.G436D	*hemi*	1.4	20/20; 20/30	20/25; 20/40
94	XLRP	*RPGR*	c.2752G>T	p.G918*	*hemi*	1.2	20/50; 20/30	20/60; 20/40

95	XLRP	*RPGR*	c.2997_2998delGG	p.E1000Gfs*78	*hemi*	1.6	20/70; 20/40	20/70; 20/50
96	XLRP	*RPGR*	c.2236_2237delGA	p.E746Rfs*23	*hemi*	1.1	20/80; 20/70	20/80; 20/70

adRP = autosomal dominant retinitis pigmentosa; arRP = autosomal recessive retinitis pigmentosa; XLRP = x-linked retinitis pigmentosa; BCVA = best-corrected visual acuity; CF = counting fingers; hemi = hemizygous; hetero = heterozygous; homo = homozygous; HM = hand motion; yrs = years.
